# Optimized Extraction of Sargahydroquinoic Acid, Major Bioactive Substance, from *Sargassum yezoense* Using Response Surface Methodology

**DOI:** 10.3390/md22120543

**Published:** 2024-12-02

**Authors:** Suhyeon Baek, Ji-Eun Bae, Yu Miao, Gahyeon Kim, Bomi Ryu, Byung-Hoo Lee, Sanggil Lee

**Affiliations:** 1 Department of Smart Green Technology Engineering, Pukyong National University, Busan 48513, Republic of Korea; bmh46750@gmail.com (S.B.); mouyu1997@gmail.com (Y.M.);; 2 Department of Food Science and Nutrition, Pukyong National University, Busan 48513, Republic of Korea; wise123@pknu.ac.kr (J.-E.B.); g6mig6mi@gmail.com (G.K.); 3 Department of Food Science & Biotechnology, Sejong University, Seoul 05006, Republic of Korea

**Keywords:** *Sargassum yezoense*, response surface methodology, antioxidant, Box–Behnken design, sargahydroquinoic acid, extraction yield

## Abstract

Sargahydroquinoic acid (SHQA), a bioactive compound found in certain *Sargassum* species, exhibits significant health benefits. This study optimized the extraction of SHQA from *Sargassum yezoense* using response surface methodology (RSM) and evaluated its antioxidant effects through in vitro and in vivo assays. A Box–Behnken design (BBD) was effectively employed to investigate the effects of incubation temperature, time, and ethanol concentration on SHQA yield, achieving a high coefficient of determination (R^2^ = 0.961). Analysis of variance (ANOVA) validated the model’s reliability (*F* = 13.86, *p* = 0.005) and highlighted ethanol concentration as a highly significant factor (*p* < 0.001). Optimal extraction conditions were identified as 52.8 °C, 8.3 h, and 74.1% ethanol. The SHQA-maximized extract (SME) contained 67.8 ± 0.6 mg SHQA/g and 25.00 ± 1.01 mg phloroglucinol equivalent/g. SME exhibited antioxidant capacity of 26.45 ± 0.66 mg and 28.74 ± 2.30 mg vitamin C equivalent/g in ABTS and DPPH assays, respectively, and 0.29 ± 0.02 mM FeSO_4_ equivalent/g in the FRAP assay. Additionally, SME at 50 µg/mL and SHQA at 1 µg/mL inhibited reactive oxygen species (ROS) generation in an H_2_O_2_-induced zebrafish model. This study presents the first optimization of SHQA extraction using RSM and demonstrates SHQA’s ROS inhibition in a zebrafish model.

## 1. Introduction

The rapid advancement of industrialization and technology has intensified environmental challenges, including global warming and pollution, highlighting the need for sustainable food solutions. Seaweed has emerged as a key player in the “green revolution” due to its carbon mitigation effect, rich nutritional profile, and diverse phytochemicals with therapeutic potential. These qualities make seaweed a sustainable resource for use in nutritious food ingredients and dietary supplements [1,2]. Globally, the seaweed market has experienced significant growth, driven by its recognized benefits and diverse applications. In particular, *Sargassum* species are widely used in Asia for both culinary and medicinal purposes, highlighting their deep cultural and health significance [3].

*Sargassum yezoense* (*S. yezoense*) is a species of brown seaweed in the family *Sargassaceae*, commonly found in temperate coastal waters, particularly in East Asia, including South Korea and Japan [4]. It is notable for its high levels of bioactive compounds, including polysaccharides, polyphenols, fucoxanthin, and meroterpenoids such as sargahydroquinoic acid (SHQA), sargachromenol, and sargaquinoic acid, which exhibit antioxidant properties and offer various health benefits [5]. Notably, sargahydroquinoic acid (SHQA), a meroterpenoid derived from *Sargassum*, has been extensively studied for its antioxidant, anti-inflammatory, anti-diabetic, and anti-aging effects on the skin [5,6,7].

Although *S. yezoense* is often consumed raw in salads, it is also used industrially in cosmetics and health supplements, such as tablets and extracts [8]. To utilize these compounds economically and efficiently, optimizing extraction conditions is essential. In particular, optimized extraction methods should target specific components of interest, considering their unique physical and chemical properties. The extraction process is influenced by multiple factors, including solvent composition, extraction time, and temperature, which all impact the yield and stability of bioactive compounds. Careful optimization of these parameters can reduce extraction time, enhance selectivity for desired compounds, and minimize the co-extraction of undesired substances [9]. Optimized extraction can also decrease solvent and energy usage, making it an environmentally friendly process [10].

Response surface methodology (RSM) is a well-established statistical tool for optimizing extraction processes, enabling the modeling and analysis of process outcomes by evaluating the interactive effects of independent variables [11]. Among the various designs in RSM, the Box–Behnken design (BBD) is especially effective, as it minimizes the number of required experiments while accurately modeling the response surface [12]. BBD has also proven effective in optimizing the extraction parameters of bioactive substances from various *Sargassum* species [13,14].

Although bioactive compounds in *Sargassum* species have been widely studied, research on optimizing extraction methods for SHQA remains limited. To address this gap, this study employed response surface methodology and Box–Behnken design (RSM-BBD) to optimize SHQA extraction conditions from *S. yezoense*. Following extraction, the antioxidant capacity of the SHQA-maximized extract (SME) was assessed, specifically its ability to mitigate oxidative stress induced by hydrogen peroxide (H_2_O_2_) exposure in a zebrafish model. This study provides a comprehensive approach to optimizing SHQA extraction, assessing its antioxidant activity, and investigating its protective effects against oxidative stress, thus establishing a foundation for future applications of SHQA in functional foods and dietary supplements.

## 2. Results

### 2.1. RSM Analysis of SHQA Extraction

SHQA content under various extraction conditions (incubation temperature, time, and ethanol concentration) is listed in Table 1. SHQA content ranged from 39.5 to 64.1 mg/g of extract under these conditions. Based on these values, a second-order polynomial equation was developed using response surface methodology (RSM) to evaluate the effects of extraction factors on SHQA content in *S. yezoense* extracts. The fitted second-order polynomial Equation (1) is as follows:

SHQA content = 60.15 − 1.09A − 2.46B + 0.44C − 1.09A^2^ − 0.30B^2^ − 7.22C^2^ − 0.96AB − 1.33AC − 1.60BC (1)

where A, B, and C represent temperature, time, and ethanol concentration, respectively.

The analysis of variance (ANOVA) results for the fitted second-order polynomial model of sargahydroquinoic acid extraction are presented in Table 2. A statistically significant *p*-value (<0.01) confirmed that the model effectively represented the experimental data. Model adequacy and quality were assessed using lack of fit (*p* > 0.05) and the coefficient of determination (R^2^). The lack of fit was insignificant (*p* = 0.25), underscoring the model’s suitability for accurately predicting variations. Regression analysis revealed a high coefficient of determination (R^2^ = 0.961), affirming the model’s accuracy and effectiveness in predicting process outcomes. As shown in Table 1, the predicted SHQA content from Equation (1) closely matched the extracted SHQA content (experimental values) under each condition, with error values from 6.3% to −4.8%. This indicates that the model reliably captured the SHQA extraction process with high reliability.

### 2.2. Effects of Extraction Temperature, Time, and EtOH Concentration

**Figure 1:**
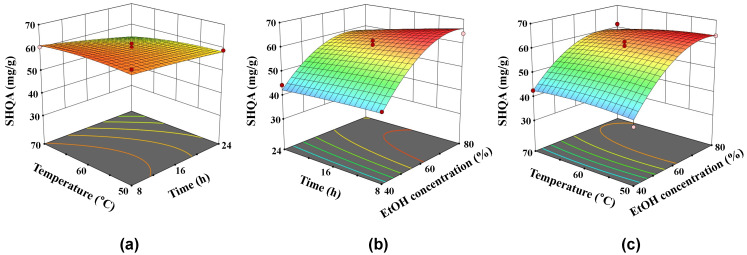
Effects of extraction variables on the sargahydroquinoic acid (SHQA) content in *S. yezoense* extracts: (**a**) temperature and time, (**b**) time and ethanol concentration, and (**c**) temperature and ethanol concentration.

The response surface plots in Figure 1a–c were generated for each factor combination, keeping the third factor constant at its intermediate value (60 °C, 16 h, and 60% EtOH, respectively). Figure 1a illustrates the interaction between incubation temperature and time on SHQA content in *S. yezoense* extract, where SHQA content decreased as temperature or time increased. This decrease was more pronounced under higher temperatures or extended times compared to milder conditions. Figure 1b shows the interaction between time and EtOH concentration, where SHQA extraction efficiency increased substantially with rising EtOH concentration at shorter times. However, at longer extraction times (around 24 h), SHQA content peaked and then declined. SHQA content exhibited a stronger decreasing trend with increased extraction time, particularly at high EtOH concentration. Figure 1c depicts the interaction between temperature and EtOH concentration, showing that SHQA extraction efficiency improved with higher EtOH concentration at lower temperatures. At higher temperatures (above 65 °C), SHQA content reached a maximum and subsequently declined. At lower EtOH concentrations, SHQA content followed a gradual curve, whereas at higher EtOH concentrations, SHQA content decreased with rising temperature.

The RSM analysis identified optimal conditions for extracting SHQA from *S. yezoense*: an extraction temperature of 52.8 °C, a time of 8.3 h, and an ethanol concentration of 74.1% (Table 3). The measured responses closely matched the predicted values, confirming the practicality of the fitted model (Table 3).

### 2.3. Antioxidant Properties of Extracts from Sargassum yezoense

Table 4 listed the antioxidant properties of SHQA-maximized extract from *S. yezoense* (SME). These results show that the *S. yezoense* extract effectively scavenges ABTS and DPPH radicals and exhibits ferrous ion-reducing power, commonly used metrics to evaluate the antioxidant capacity of natural extracts.

### 2.4. Protective Effect of SME and SHQA Against H_2_O_2_-Induced Oxidative Stress

**Figure 2:**
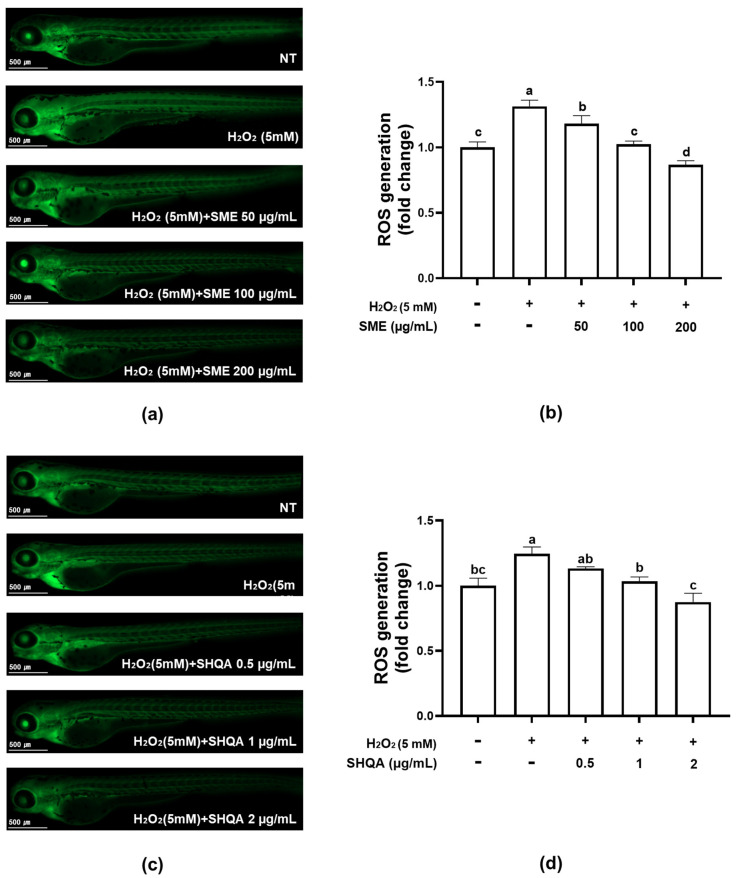
The protective effect of sargahydroquinoic acid (SHQA)-maximized extracts from *S. yezoense* (SME) (**a**,**b**) and pure SHQA (**c**,**d**) on H_2_O_2_-induced ROS generation was evaluated in a zebrafish model. Panels (**a**) and (**c**) show microscopic fluorescence images of zebrafish embryos, while panels (**b**) and (**d**) display the corresponding relative fluorescence intensities, representing ROS levels. “NT” indicates the untreated control group. Different letters above the bar chart indicate significant differences (*p* < 0.05).

Zebrafish are extensively used in bioactivity screening studies due to their short lifespan and genomic similarities to mammals [15,16]. Their morphological and molecular similarities to tissue and organs in other vertebrates, including humans, enhance their values as model organisms. As a result, the zebrafish model is frequently employed in in vivo studies to assess antioxidant capacity [17]. Figure 2 shows the in vivo evaluation of the protective effects of SME and SHQA against H_2_O_2_-induced oxidative stress using the zebrafish model. H_2_O_2_ induces cell damage through the generation of reactive oxygen species (ROS), leading to oxidative stress [17]. ROS production in zebrafish embryos treated with H_2_O_2_ was assessed using oxidation-sensitive DCFH-DA staining, which measures oxidative activity within cells. The fluorescence intensity from this staining indicates ROS levels.

The results showed that H_2_O_2_-treated embryos without SME treatment exhibited significantly higher fluorescence intensity compared to the non-treated group (NT), indicating ROS generation due to H_2_O_2_ treatment. In contrast, ROS generation was inhibited in a dose-dependent manner with SME (50–200 µg/mL) and SHQA (0.5–2 µg/mL). With a concentration of 50 µg/mL of SME and 1 and 2 µg/mL of SHQA, fluorescence intensity by oxidative stress was reduced to levels comparable to the NT group (Figure 2b,d). This suggests that SME and SHQA effectively scavenge ROS and functions as a protective agent.

## 3. Discussion

*Sargassum* species have garnered attention for their high-value extracts due to the presence of bioactive compounds such as phenolic compounds, polysaccharides, and meroterpenoids. These compounds demonstrate a range of physiological effects, including antioxidant, anti-inflammatory, anti-diabetes, and anti-aging properties. For instance, Kim et al. [18] studied the anti-diabetic effect of *S. yezoense* in mice, while Lim et al. [6] showed that *S. serratifolium* suppressed oxidative stress induced by tert-butyl hydroperoxidase in HepG2 cells. Joung et al. [7] reported that SHQA derived from *S. macrocarpum* attenuated inflammatory responses, and more recently, Park et al. [19] explored the anti-inflammatory effects of SHQA from *S. yezoense*.

The extraction of bioactive compounds from *S. yezoense* requires careful consideration of parameters such as temperature, time, and solvent concentration, as these factors substantially affect both the yield and antioxidant properties of the extracted compounds. Heo et al. [20] found that methanolic extracts from *S. fulvellum* and *S. horneri* exhibited higher total phenolic content (TPC) when extracted at 70 °C compared to 20 °C, whereas for aqueous extracts, TPC was higher at 20 °C than at 70 °C. Therefore, it is essential to consider the interactive effects of these parameters. RSM proves to be an effective tool for optimizing these conditions. This study demonstrates that the RSM model, integrating three independent variables (temperature, time, and ethanol concentration), accurately explains and predicts SHQA content. The statistically significant *p*-values suggest that the model reliably represents the experimental data, while the lack-of-fit test confirms the model’s suitability for the extraction process. Furthermore, the high coefficient of determination (R^2^ = 0.961) supports the robustness of the model in predicting SHQA content across varying extraction conditions. The low error values observed between predicted and experimental data indicate that the model is accurate and practical for optimizing SHQA extraction conditions in *S. yezoense*.

The extraction temperature generally has a significant impact on extraction efficiency. As the extraction temperature increases, the solvent’s dissolving capacity improves, enhancing the permeability of the cell walls of brown algae and allowing the target compounds to migrate more quickly. However, the extraction temperature must be finely adjusted in relation to the extraction time. This is because many bioactive compounds, particularly SHQA, are heat-sensitive, but if the temperature is optimal, prolonged extraction can lead to the degradation of the compound. Therefore, it is crucial to establish optimal extraction conditions that simultaneously consider both extraction temperature and time. Previous studies also have shown that ideal extraction temperatures typically range from 40 °C to 80 °C, with higher temperatures often enhancing the solubility of the compounds. However, exceeding the optimal temperature can damage heat-sensitive components [21,22].

The RSM analysis identified optimal extraction conditions for SHQA as 52.8 °C, 8.3 h, and 74.1% ethanol. These conditions align with ideal extraction parameters proposed in prior studies [23,24] for temperature (50–60 °C), time (6–9 h), and EtOH concentration (around 70%). These conditions help prevent degradation of heat-sensitive compounds, balance extraction efficiency and compound stability, and maximize phenolic solubility while excluding non-polar impurities. The RSM results also indicated that SHQA extraction efficiency decreased at higher temperatures, potentially due to heat-induced degradation or structural changes in SHQA. Optimal extraction times vary widely: while some studies suggest shorter durations (30 min to 2 h) suffice for certain compounds, others require up to 24 h for maximal yield [25,26]. However, prolonged extraction risks degrading heat-sensitive compounds, highlighting the need for a temperature–time balance to achieve optimal extraction. Baek et al. [27] found that a chloroform fraction rich in fucoxanthin, a fat-soluble bioactive compound found in kelp, exhibited stability with only about 10% degradation after 12 h at 55 °C, but approximately 50% degradation after 24 h. At 75 °C, however, about 70% degradation was observed within just 6 h, indicating a significant reduction in the stability of the substance with increasing temperature. This suggests that an extraction time of 8.3 h at 52.8 °C in the current study ensured optimal stability of SHQA during SME extraction. In particular, ethanol extracts are thought to contain a wider range of compounds compared to the chloroform fraction and may have also helped prevent the degradation of SHQA during extraction. Additionally, the ethanol concentration showed the greatest impact on the extraction yield of SHQA. Lim et al. [28] found that ethanol was the most efficient solvent for extracting SHQA from *S. serratifolium*. Given that binary solvents composed of water and organic solvents, such as ethanol, have been shown to be effective for extracting phenolics or terpenoids from seaweeds due to the increased dielectric constant provided by water [29], selecting the appropriate ethanol concentration is crucial for optimizing the extraction process. In this regard, the selection of extraction parameters should be tailored to the specific bioactive compounds of interest, which can be achieved through RSM.

Oxidative damage results from an imbalance between ROS levels and antioxidant capacity within organisms. ROS are highly reactive oxygen derivatives that include free radicals, such as hydroxyl radicals, as well as non-radical molecules like H_2_O_2_ [30]. Phenolic compounds are well known for their ability to donate hydrogen atoms and neutralize free radicals, thereby reducing oxidative stress in biological systems [31]. The high phenolic content of SME highlights phenolics as key contributors to antioxidant activity in seaweed extracts [32]. The enhanced radical scavenging efficacy of SME aligns with previous studies that highlight the remarkable antioxidant properties of *Sargassum* species [33]. SME exhibited higher phenolic content and antioxidant capacity compared to other *Sargassum* species. For instance, Park et al. [34] reported that the methanol extract of *S. thunbergii* contained 1.16 mg PGE/g. Similarly, Zhao et al. [35] found that the antioxidant activity of freeze-dried *Sargassum fusiforme* was 1.2 mg VCE/g and 0.42 mg VCE/g on ABTS and DPPH assays, respectively.

These properties could be attributed to polyphenolic compounds containing hydroquinones like SHQA [36]. The effect of SHQA as antioxidants was proved through an in vivo system of zebrafish. Joung et al. [37] found that the ethanol extract of *S. serratifolium* and its primary bioactive compound, SHQA, exhibited potent anti-inflammatory effects and strong reactive oxygen species (ROS) scavenging activities. As a powerful antioxidant, SHQA likely reduces ROS through mechanisms similar to those reported by Joung et al. [7], who demonstrated that SHQA reduces inflammation and oxidative stress, mainly by inhibiting the NF-κB pathway and activating the Nrf2/HO-1 pathway. Specifically, in the NF-κB pathway, SHQA blocks critical steps that would otherwise elevate inflammation, thereby decreasing pro-inflammatory cytokine levels. Concurrently, SHQA activates Nrf2, which enhances the expression of HO-1, an antioxidant enzyme that lowers ROS levels, thus protecting cells from oxidative damage. In our zebrafish study, SME and SHQA potentially reduced ROS generation, which may enhance cellular defenses against oxidative damage induced by H_2_O_2_, thereby demonstrating their antioxidant properties in vivo. This highlights the protective effects of SHQA against H_2_O_2_-induced oxidative stress, further establishing its significance in health-related applications and its potential role in mitigating oxidative damage. This study also emphasizes the effectiveness of RSM in optimizing extraction conditions for SHQA from *S. yezoense*, showcasing its ability to improve extraction efficiency while preserving bioactive compounds. The substantial antioxidant properties of SHQA combined with the high phenolic content of *S. yezoense* underscore the potential of this seaweed species for health-related applications. However, the time and location of *Sargassum yezoense* harvest may affect the concentration of bioactive compounds, such as SHQA. Further research is needed to evaluate these factors and their impact on extraction efficiency and bioactivity.

## 4. Materials and Methods

### 4.1. Materials

Formic acid, Folin–Ciocâlteu phenol reagent, 2,2′-azino-bis (3-ethylbenzthiazoline-6-sulfonic acid) (ABTS) diammonium, 2,2′-azobis-(2-amidinopropane) HCl (AAPH), ascorbic acid, potassium phosphate monobasic, potassium phosphate dibasic, sodium acetate, glacial acetic acid, HCl, TPTZ (2,4,6-tri[2-pyridyl]-s-triazine) iron (III) chloride, and FeSO_4_7H_2_O were purchased from Roche (Roche, Basel, Switzerland).

### 4.2. Sample Preparation and Extraction

The *S. yezoense* was cultivated in February on Jeju Island (Republic of Korea). After washing 500 g of *S. yezoense* using tap water for 30 min 3 times, the *S. yezoense* was frozen at −80 °C and freeze-dried, then ground using a blender. For RSM analysis, 1 g of a ground sample was extracted with 40 mL of various concentrations of EtOH under different temperatures and times. The extracted samples were filtered using a 0.45 µM PVDF syringe filter and stored at −50 °C for further studies.

### 4.3. Optimization of Extraction Conditions

The optimization of extraction conditions to achieve the highest extraction yield of SHQA from *S. yezoense* was conducted using the RSM-based BBD method.

#### 4.3.1. Quantification of SHQA by High-Performance Liquid Chromatography (HPLC)

Chromatographic analysis was performed using HPLC equipped with a SUPERSIL ODS-III column (250 mm × 4.6 mm, 5 μm particle size). The SHQA was detected at 230 nm, with a UV-photodiode array (PDA) detector (Dionex Corp, Sunnyvale, CA, USA) scanning the wavelength range of 190–700 nm. Deionized water (DIW) containing 0.1% (*v*/*v*) formic acid (solvent A) and HPLC-grade acetonitrile containing 0.1% (*v*/*v*) formic acid (solvent B) were used for gradient elution. The flow rate was set to 1.0 mL/min, and the injection volume for both samples and standard compounds (purified SHQA, as described in Section 4.6) was 10 μL. The analysis was performed with the column temperature maintained at 35 °C and the autosampler temperature at 25 °C. The gradient elution profile was as follows: 0 min (90:10), 3 min (90:10), 8 min (23:77), 10 min (23:77), 28 min (18:82), 32 min (0:100), 35 min (0:100), 40 min (90:10), and 42 min (90:10). Using this gradient elution, calibration curves and linear equations of peak area versus concentration were determined for the SHQA content (mg/g).

#### 4.3.2. Experimental Design

An RSM-based BBD method with three levels was employed to optimize the extraction variables (Table 1). The independent variables included temperature (A, 50–70 °C), incubation time (B, 8–24 h), and ethanol concentration (C, 40–80%). The coded and actual values for the BBD of these independent variables are presented in Table 1. The predicted response was determined using a second-order polynomial model, and the response surface analysis is described by the following Equation (2).

Y = b_0_ + b_1_X_1_ + b_2_X_2_ + b_3_X_3_ + b_12_X_1_X_2_ + b_13_X_1_X_3_ + b_23_X_2_X_3_ + b_11_ + b_22_ + b_33_ (2)

According to the results from RSM, *S. yezoense* was extracted using 40 mL of 74.1% ethanol at 52.8 °C for 8.3 h (Table 3), under conditions devoid of light and oxygen. The extracts obtained under these conditions are referred to as SHQA-optimized extracts from *S. yezoense* (SME).

### 4.4. Total Phenolic Content (TPC)

The total phenolic content was determined through a colorimetric assay utilizing Folin–Ciocâlteu reagent. Samples diluted tenfold (10 μL) were combined with 130 μL of deionized water (DIW) in a 96-well microplate. Folin–Ciocâlteu reagent (10 μL) was then added, and the mixture was allowed to react at room temperature for 6 min. To initiate color development, 100 μL of 7% Na_2_CO_3_ solution was added, and after 90 min, the absorbance was measured at 750 nm. Phloroglucinol was used as the standard, and a phloroglucinol solution ranging from 10 to 100 µg/mL was prepared for the standard curve. The results are reported as milligrams of phloroglucinol equivalent (PGE) per gram of sample.

### 4.5. Total Antioxidant Capacity (TAC) by ABTS, DPPH, and FRAP Assays

#### 4.5.1. ABTS Assay

The ABTS assay was based on the method of van den Berg et al. [38]. Briefly, the ABTS reagent was prepared by combining 1.0 mM AAPH, 2.5 mM ABTS, and phosphate-buffered saline. After mixing AAPH and ABTS, ABTS radicals were generated by heating the mixture in an 80 °C water bath for 40 min. The resulting radical solution was then filtered using a 0.45 μm PVDF syringe filter. Diluted SME (10 μL) was mixed with the 240 μL of ABTS reagent and incubated at 37 °C for 10 min. The absorbance was measured at 734 nm. Ascorbic acid (vitamin C) was used as the standard, and a vitamin C solution ranging from 10 to 100 µg/mL was prepared for the standard curve. The TAC by ABTS is expressed as mg vitamin C equivalent (VCE)/g.

#### 4.5.2. DPPH Assay

The DPPH assay was conducted according to the method developed by Brand-Williams et al. [39]. The assay was based on the change in absorbance of the sample. The DPPH reagent was prepared by dissolving 7.89 mg of DPPH (2,2-diphenyl-1-picrylhydrazyl) in 80% EtOH. The 245 μL of DPPH reagent was mixed with 5 μL of diluted SME. After incubating the mixture for 30 min at room temperature, the absorbance was measured at 510 nm. Ascorbic acid (vitamin C) was used as the standard, and a vitamin C solution ranging from 10 to 100 µg/mL was prepared for the standard curve. The TAC determined by the DPPH assay is expressed as mg VCE/g.

#### 4.5.3. Ferric Reducing Power (FRAP) Assay

The FRAP assay was conducted using a slightly modified method of Baek et al. [27]. Briefly, a FRAP reagent was prepared by mixing 10 mM TPTZ, 20 mM FeC_3_ solution, and 300 mM of acetate buffer (pH 3.6) with DIW in a 1:1:10:1.2 ratio. For the measurement, 7.5 μL of diluted SME was added to 250 μL of the FRAP reagent and incubated at 37 °C for 4 min. After incubation, the absorbance was measured at 593 nm. Iron (II) sulfate heptahydrate (FeSO_4_) was used as the standard, and a standard solution ranging from 10 to 100 µg/mL was prepared for the standard curve. The reducing power of the SME sample is expressed as mM ferrous sulfate equivalent (FeSO_4_) equivalent/g.

### 4.6. Purification Method for SHQA

For SHQA purification, 10 g of freeze-dried *S. yezoense* was extracted with 50 mL of dichloromethane (DCM) and MeOH mixture (2:1) using sonication for 1 h. The extract was then mixed with 50 mL of DIW, centrifuged at 4000 rpm for 5 min, and the supernatant discarded to remove polysaccharides or other water-soluble substances. This step was repeated, and the collected precipitate was evaporated at 35 °C. The residue was re-dissolved in 5 mL of DCM and injected into 80 g of RediSep normal phase silica column (Teledyne Isco, Inc., Lincoln, NE, USA). Separation of SHQA was performed using CombiFlash medium-pressure liquid chromatography (MPLC) with a mobile phase consisting of A: hexane and B: chloroform–hexane–acetone–methanol (62:28:9:1). The column was equilibrated with 160 mL with hexane at a flow rate of 20 mL/min. The mobile phase A was run for 5 min, followed by a gradient increase to 100% of mobile phase B for 30 min. Fractions were evaporated and dissolved in acetonitrile (ACN) with 1% acetic acid, then filtered through a 0.45 μm PVDF filter in preparation for HPLC.

HPLC conditions were as follows. Waters Semi-Prep ODS column (10 μm, 10 × 250 mm); gradient elution performed using an A eluent (0.1 % formic acid in water, *v*/*v*) and a B eluent (0.1 % formic acid in ACN, *v*/*v*) with the following linear gradient combinations: at 0 min, 70% B; at 2 min, 70% B; at 8 min, 78% B; at 13 min, 78% B; at 25 min, 85% B; at 28 min, 85% B; at 29 min, 100% B; at 31 min, 100% B; and at 35 min, 70% B. Flow rate was 2.0 mL/min, and 60 μL sample was injected into the column. The effluent was monitored at 230 nm for SHQA. Fractions were evaporated under nitrogen gas (Figure S1).

### 4.7. Estimation of Intracellular ROS Generation in Zebrafish Embryos

Wild-type adult zebrafish were purchased from a commercial dealer (Greenfish, Seoul, Republic of Korea) and maintained in a 3.5 L acrylic tank at 28.5 ± 1 °C with a 14/10 h light/dark cycle. They were fed artemia flakes twice daily. Embryos were obtained from natural spawning stimulated by light and were randomly distributed into 12-well plates at 7–9 h post-fertilization (hpf), with 15 embryos per well in 1 mL of embryo medium. Embryos were treated with SME at concentrations of 0, 50, 100, and 200 µg/mL and purified SHQA at concentrations of 0, 0.5, 1, and 2 µg/mL for 1 h, followed by the induction of oxidative stress using 5 mM H_2_O_2_. Survival was monitored daily throughout the experiment. Heartbeat rates were measured at 2 days post-fertilization (dpf) for 3 min using a microscope, with results expressed as average heartbeats per minute. Zebrafish larvae at 3 dpf were treated with 2,7-dichlorofluorescein diacetate (DCFH-DA) for 1 h, then washed twice with fresh embryo medium. After anesthetizing the larvae with 0.003% ethyl 3-aminobenzoate methane sulfonate (Sigma-Aldrich, St. Luis, MO, USA), reactive oxygen species (ROS) production was assessed through fluorescence microscopy (BioTek Cytation 5 Cell Imaging Multimode Reader). The fluorescence intensity was quantified using ImageJ software (2.14.0 version). This study received IRB approval from Jeju National University (2022-0005; approval date: 17 April 2023).

### 4.8. Statistical Analysis

All experiments were conducted in triplicate, differences in mean values of results were tested by analysis of variance (ANOVA) and determination of significance of difference among samples using *p*-values obtained by Tukey’s HSD multiple rank test (*p* = 0.05).

## 5. Conclusions

This study highlights the importance of optimizing extraction conditions for maximizing the antioxidant capacity of *Sargassum yezoense*, which could be approached with RSM. By carefully selecting extraction conditions, it is possible to preserve heat-sensitive compounds and ensure efficient extraction of phenolic compounds, which are key to the antioxidant properties of extracts. In this study, 52.8 °C, 8.3 h, and 74.1% ethanol were the optimal conditions. The results of total phenolic content and total antioxidant capacity support *Sargassum* serving as an effective antioxidant source. The antioxidant capacity of *S. yezoense* was found to be particularly pronounced due to the presence of SHQA in the extract, as demonstrated by significant reductions in oxidative stress in zebrafish model experiments. From these results, this study offers valuable insights for future applications in functional food and nutraceutical industries.

## Figures and Tables

**Table 1:** Sargahydroquinoic acid (SHQA) content and predicted values with extraction variables.

Run	Independent Variables			Dependent Variables		
	Temperature	Time	Ethanol Concentration	SHQA Content (mg/g)		Error ^a^ (%)
				Experimental Values	Predicted Values	
1	50 °C	16 h	40%	39.5	42.2	6.3
2	70 °C	24 h	60%	52.2	54.2	3.8
3	70 °C	16 h	40%	42.7	42.6	−0.1
4	60 °C	24 h	40%	44.4	42.3	−4.8
5	60 °C	24 h	80%	57.4	58.0	1.0
6	70 °C	16 h	80%	61.5	58.8	−4.5
7	60 °C	16 h	60%	61.9	60.1	−3.0
8	60 °C	8 h	80%	64.1	66.1	3.1
9	60 °C	16 h	60%	60.3	60.1	−0.2
10	50 °C	24 h	60%	59.0	58.4	−1.1
11	50 °C	16 h	80%	63.7	63.7	0.0
12	70 °C	8 h	60%	60.5	61.1	1.0
13	60 °C	16 h	60%	58.2	60.1	3.2
14	60 °C	8 h	40%	44.7	44.1	−1.4
15	50 °C	8 h	60%	63.4	61.3	−3.4

^a^ Error (%) = 100 − (experimental value/predicted value × 100).

**Table 2:** Analysis of variance (ANOVA) for the fitted second-order polynomial model of sargahydroquinoic acid extraction.

Source *	DF ^a^	Adj SS ^b^	Adj MS ^c^	*F*-Value	*p*-Value
Model	9	985.07	109.45	13.86	0.005
Linear					
A	1	9.57	9.57	1.21	0.321
B	1	48.29	48.29	6.12	0.056
C	1	712.25	712.25	90.19	0.000
Squares					
A*A	1	4.38	4.38	0.55	0.490
B*B	1	0.33	0.33	0.04	0.847
C*C	1	192.51	192.51	24.38	0.004
2-way interactions					
A*B	1	3.72	3.72	0.47	0.523
A*C	1	7.11	7.11	0.90	0.386
B*C	1	10.24	10.24	1.30	0.307
Residual	5	39.49	7.90	3.16	
Lack of fit	3	32.61	10.87		0.249
Pure error	2	6.87	3.44		
Cor total	14	1024.56			

^a^ Degrees of freedom. ^b^ Adjusted sum of squares. ^c^ Adjusted mean square. * A, B, and C represent temperature, time, and ethanol concentration, respectively.

**Table 3:** Optimized conditions for sargahydroquinoic acid (SHQA) extraction and corresponding response values.

**Optimum Condition**	**Temperature**		**Time**		**EtOH Concentration**	
	52.8 °C		8.3 h		74.1%	
**Response** **(SHQA content, mg/g)**	**Predicted** **value**	**Experimental** **value**		**95% CI ^a^**		**95% PI ^b^**
	66.62	67.8 ± 0.60		(60.80, 72.44)		(46.93, 86.30)

^a^ Confidence interval; ^b^ Prediction interval.

**Table 4:** Total phenolic content (TPC) and total antioxidant capacity by ABTS, DPPH, and FRAP assay of sargahydroquinoic acid (SHQA)-maximized extracts from *Sargassum yezoense* (SME).

Sample	TPC (mg PGE/g)	ABTS (mg VCE/g)	DPPH (mg VCE/g)	FRAP (mM FeSO_4_/g)
SME	25.00 ± 1.01	26.45 ± 0.66	28.74 ± 2.30	0.29 ± 0.02

## Data Availability

Data are contained within the article or Supplementary Materials. Further inquiries can be directed to the corresponding author.

## Supplementary Materials

The following supporting information can be downloaded at https://www.mdpi.com/article/10.3390/md22120543/s1. Figure S1. Chromatograms of SME and SHQA for standard, spectrum of SHQA, and chemical structure of SHQA.
