# Supplementary material for: Optimized Extraction of Sargahydroquinoic Acid, Major Bioactive Substance, from Sargassum yezoense Using Response Surface Methodology
Source: Mar Drugs. 2024 Dec 2;22(12):543. doi: 10.3390/md22120543 (PMC11676218; doi:10.3390/md22120543)
Supplement: Supplementary file 1 [file marinedrugs-22-00543-s001.zip › marinedrugs-3318075-supplementary.pdf]

Optimized Extraction of Sargahydroquinoic Acid, Major Bioactive Substance, from *Sargassum yezoense* Using Response Surface Methodology

Suhyeon Baek [1,†], Ji-Eun Bae [2,†], Yu Miao [1], Gahyeon Kim [2], Bomi Ryu [1,2], Byung-Hoo Lee [3,*] and Sanggil Lee [1,2,*]

[1] Department of Smart Green Technology Engineering, Pukyong National University, Busan 48513, Republic of Korea; bmh46750@gmail.com (S.B.); mouyu1997@gmail.com (Y.M.); bmryu@pknu.ac.kr (B.R.)
[2] Department of Food Science and Nutrition, Pukyong National University, Busan 48513, Republic of Korea; wise123@pknu.ac.kr (J.-E.B.); g6mig6mi@gmail.com (G.K.)
[3] Department of Food Science & Biotechnology, Sejong University, Seoul 05006, Republic of Korea
* Correspondence: bhlee@sejong.ac.kr (B.-H.L.); sglee1125@pknu.ac.kr (S.L.); Tel.: +82-02-3408-3228 (B.-H.L.); +82-051-629-5842 (S.G.).
† These authors contributed equally to this work.

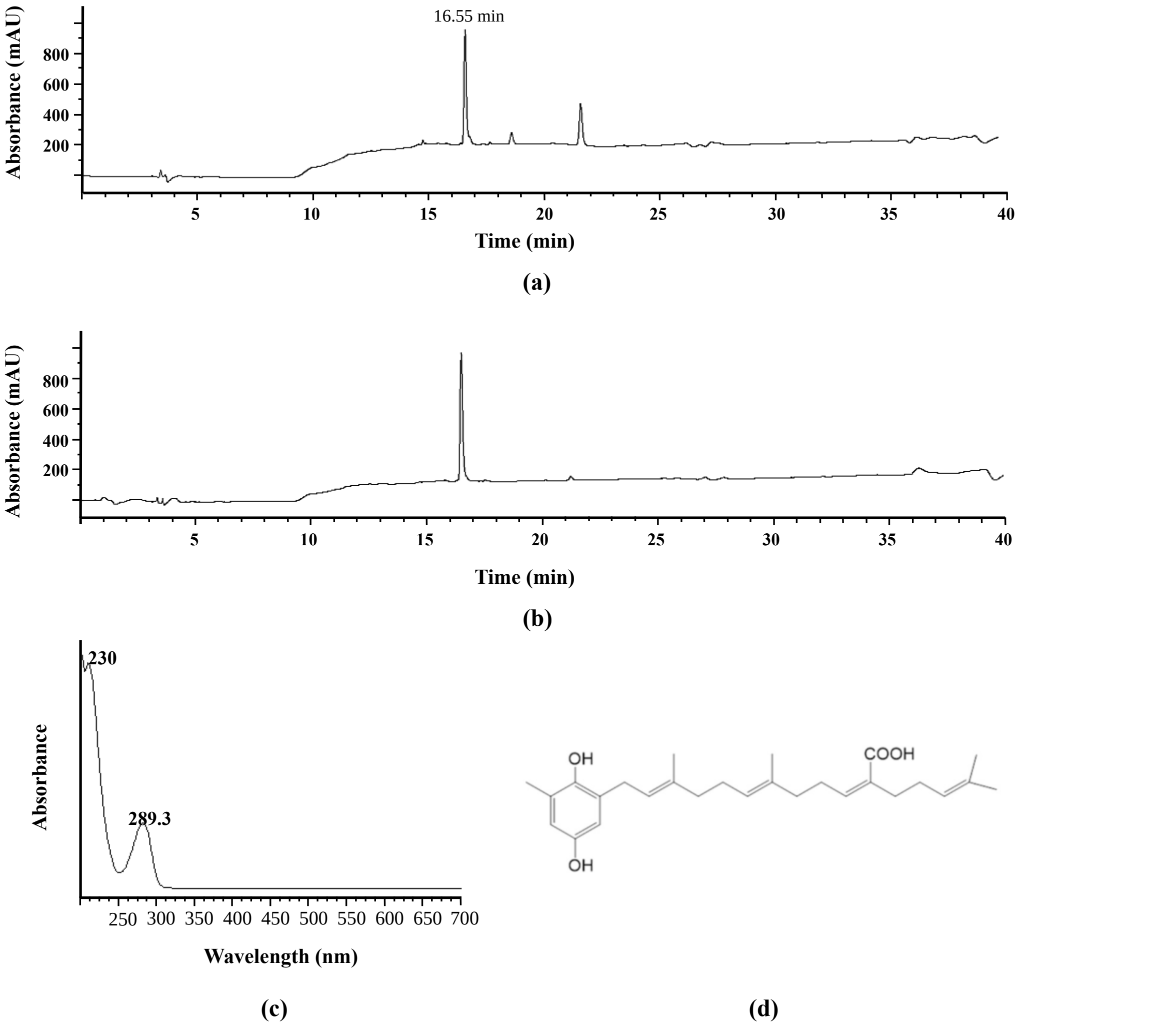

Figure S1. Chromatograms of (a) SME and (b) SHQA for standard, (c) spectrum of SHQA, and (d) chemical structure of SHQA.
